# The Use of Digital Technology to Enhance Student Learning Experience in Preclinical Operative Dentistry

**DOI:** 10.1002/jdd.13936

**Published:** 2025-05-12

**Authors:** Daria Ghorbanpoor, Jordan Asnes, Andrea Mantesso, Clarissa S. G. da Fontoura

**Affiliations:** ^1^ School of Dentistry University of Michigan Ann Arbor Michigan USA; ^2^ Department of Cariology, Restorative Sciences and Endodontics School of Dentistry, University of Michigan Ann Arbor Michigan USA

**Keywords:** educational technology | simulation laboratory preparation | student computer‐assisted instruction | undergraduate dental education

## 1 Problem

The dental curriculum is designed to support the progression of psychomotor skills, hand‐eye coordination, and cognitive understanding. Simulation laboratory education helps address these challenges, offering students opportunities to practice their skills. However, traditional methods may fail to provide the detailed and interactive visualizations necessary for mastering concepts [1]. The implementation of three‐dimensional (3D) technology has proven successful for dental students, positively impacting learning [2, 3, 4]. This technology enhances traditional methods by improving students' ability to analyze exercises and engage with the material through interactive experiences [2, 4, 5]. However, no study has investigated students' motivation for using this technology as a learning tool.

## 2 Solution

We aimed to use 3D technology in preclinical operative dentistry courses to enhance visual learning and rubric comprehension compared to traditional learning aids. Additionally, we assessed motivation to use the technology.

**FIGURE 1:**
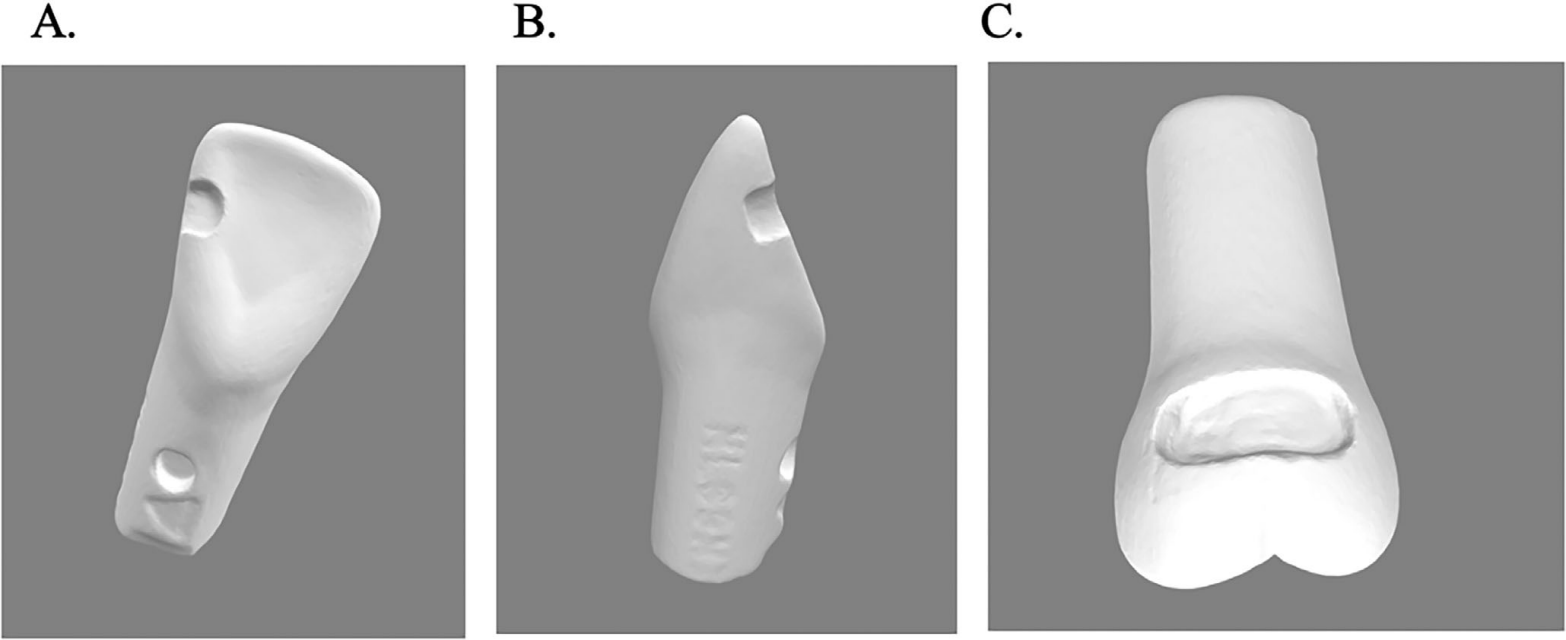
The provided images depict a TRIOS three‐dimensional (3D) scan capturing Class III and Class V dental preparations utilized for instructional purposes in operative dentistry simulation laboratory classes. Image A illustrates the lingual perspective of an exemplary Class III preparation on a maxillary central incisor. Image B offers a mesial view of a Class III preparation on a maxillary central incisor. Image C presents a buccal view of a Class V preparation executed by a student on a maxillary molar.

The TRIOS system was used to 3D‐scan five previously graded classes III and V student preparations. These scans were presented to students during class, along with detailed explanations (Figure 1A–C). An ideal preparation sample was also presented alongside these scans for comparison. Following the course rubrics, students assessed the preparations before disclosing the faculty's grading. Subsequently, the faculty's grades were revealed, accompanied by comprehensive explanations.

We conducted a digital survey to evaluate students' perceived ability in formative learning. After the presentations, we surveyed 109 students to assess their perceived comprehension and knowledge of the course expectations. The survey included 11 multiple‐choice questions and three open‐ended questions.

The Chi‐square test for independence was then used to analyze the data, as this test is suitable for comparing categorical data—expressly, the distribution of responses (e.g., “Strongly Agree” and “Somewhat Agree”) across different survey questions.

## 3 Results

A survey of 109 students in a simulation laboratory found that 51.1% somewhat agreed and 42.4% strongly agreed that 3D scans significantly aided their learning (Table 1). Regarding rubric comprehension, 44.6% somewhat agreed, and 50% strongly agreed that examining the scans improved their understanding. Only 2.2% held neutral, and 3.3% had mildly negative views on rubric understanding.

**TABLE 1:** Results from the survey of 109 students in a simulation laboratory.

Survey questions	Strongly Disagree (%)	Somewhat Disagree (%)	Neither Agree nor Disagree (%)	Somewhat Agree (%)	Strongly Agree (%)	Total responses
I enjoyed seeing the 3D‐scanned preparations	2.2	2.2	4.4	42.4	48.9	92.0
It was difficult to learn from these 3D scans	20.7	45.7	17.4	14.1	2.2	92.0
Viewing these 3D scans aided my learning	1.1	0.0	5.4	51.1	42.4	92.0
Examining these scans improved my understanding of the rubrics	0.0	3.3	2.2	44.6	50.0	92.0
Observing these 3D scans did not further motivate me in preclinic	26.1	37.0	28.3	6.5	2.2	92.0
Viewing these 3D scanned preparations helped to clarify preclinic expectations	0.0	2.2	4.4	58.7	34.8	92.0
These 3D scans improved my ability to identify preparation guidelines	0.0	2.2	3.3	54.4	40.2	92.0
I did not feel engaged while working with the 3D scanned preparations and restorations	27.2	28.3	26.1	14.1	4.4	92.0
These 3D scans allowed me to better understand how the practical exam is graded	0.0	1.1	6.6	48.4	44.0	91.0
Analyzing these 3D scanned preparations and restorations was too time‐consuming	22.8	33.7	17.4	16.3	9.8	92.0
Reviewing these 3D scans allowed me to identify common preparation mistakes	1.1	0.0	6.5	45.7	46.7	92.0

Despite positive feedback, students suggested improvements, such as increasing access to the scans (17% requested more access), presenting the scans earlier in the course, and providing more examples. Some felt that the scan presentations took away valuable time from hands‐on practice in the simulation lab, and we acknowledge that the method is not an all‐inclusive preparation evaluator.

**FIGURE 2:**
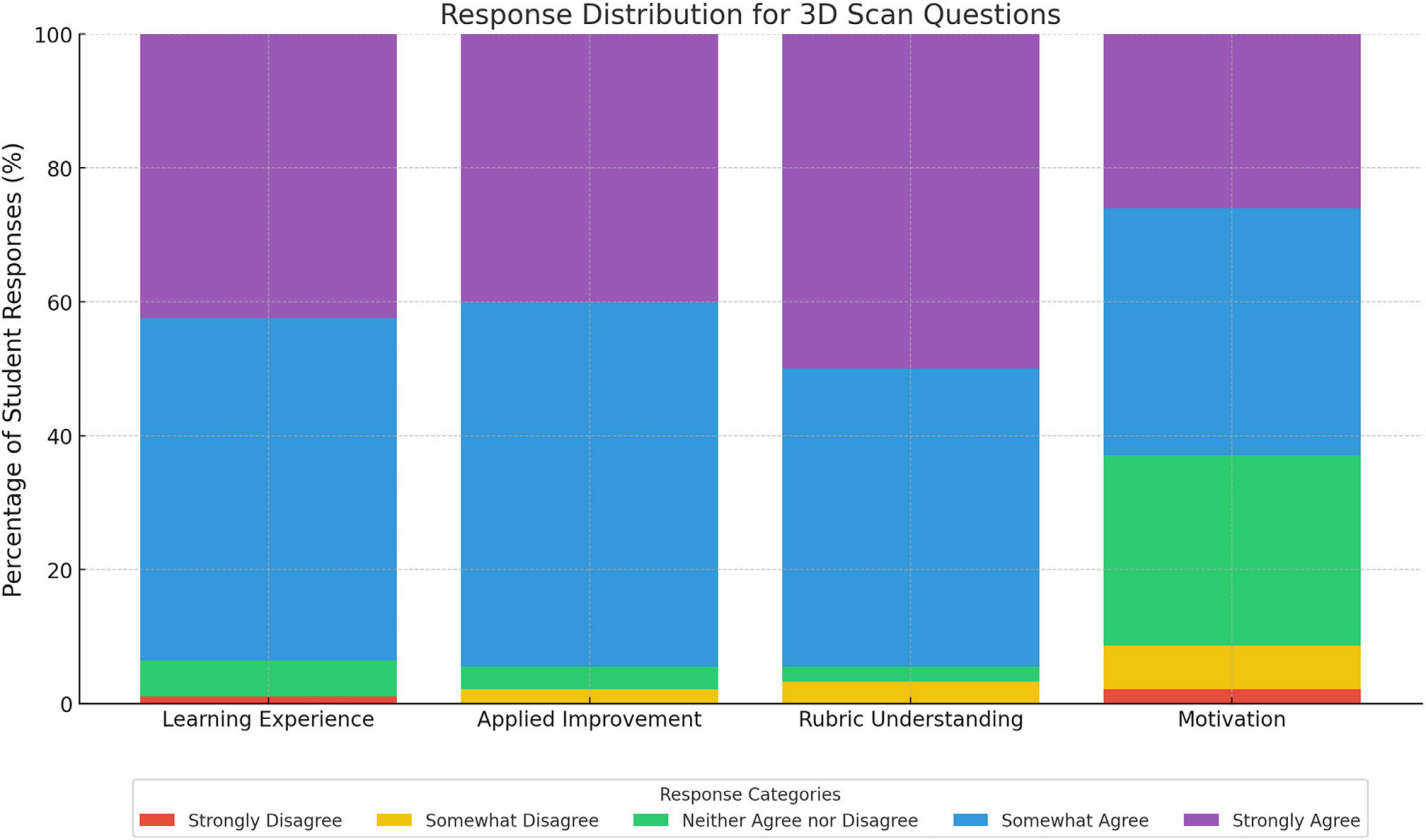
This stacked bar chart illustrates the distribution of student responses to questions about the perceived effectiveness of three‐dimensional (3D) scans in enhancing learning, applied improvement, rubric understanding, and motivation. The stacked bar chart displays student responses to four specific survey questions, each aligned with a question category. Learning experience corresponds to the survey question: *“Viewing these 3D scans aided my learning.”* Applied improvement reflects responses to: *“These 3D scans improved my ability to identify preparation guidelines.”* Rubric understanding relates to: *“Examining these scans improved my understanding of the rubrics.”* Motivation corresponds to the statement: *“Observing these 3D scans did not further motivate me in preclinic.”* Each bar represents a question category, with different colors indicating the percentage of students selecting each response (e.g., "Strongly Agree" and "Somewhat Disagree"). Most students responded positively to the impact of 3D scans on learning, applied improvement, and rubric understanding, as reflected by the larger proportions of "Somewhat Agree" and "Strongly Agree" responses. In contrast, motivation shows a broader spread of responses, with fewer students strongly agreeing that the 3D scans motivated them in preclinic practice.

Statistical analysis revealed significant differences in how students rated learning, rubric understanding, and motivation when 3D technology is used for preclinical courses. While the scans were effective for perceived learning and skill development, they did not significantly motivate students. The *p*‐values for learning vs. motivation (*p* < 0.0001), applied improvement vs. motivation (*p* < 0.0001), and rubric understanding vs. motivation (*p* < 0.0001) indicate a stark contrast in motivation levels (Figure 2).

This suggests that while students recognize the educational value of the scans, they were not particularly effective in motivating students. Instructors could focus on making the 3D scans more interactive, immersive, or rewarding to increase student motivation alongside their learning.

## Author Contributions

All authors gave final approval and agreed to be accountable for all aspects of the work.

## Conflicts of Interest

The authors declare no conflicts of interest.
